# Muscle Synergies and Clinical Outcome Measures Describe Different Factors of Upper Limb Motor Function in Stroke Survivors Undergoing Rehabilitation in a Virtual Reality Environment

**DOI:** 10.3390/s21238002

**Published:** 2021-11-30

**Authors:** Lorenza Maistrello, Daniele Rimini, Vincent C. K. Cheung, Giorgia Pregnolato, Andrea Turolla

**Affiliations:** 1 Laboratory of Rehabilitation Technologies, IRCCS San Camillo Hospital, 30126 Venice, Italy; lorenza.maistrello@hsancamillo.it (L.M.); giorgia.pregnolato@hsancamillo.it (G.P.); andrea.turolla@hsancamillo.it (A.T.); 2 Medical Physics Department—Clinical Engineering, Salford Care Organisation, Salford M6 8HD, UK; 3 School of Biomedical Sciences, The Chinese University of Hong Kong, Hong Kong, China; vckc@cuhk.edu.hk

**Keywords:** muscle synergies, sEMG, stroke, factor analysis

## Abstract

Recent studies have investigated muscle synergies as biomarkers for stroke, but it remains controversial if muscle synergies and clinical observation convey the same information on motor impairment. We aim to identify whether muscle synergies and clinical scales convey the same information or not. Post-stroke patients were administered an upper limb treatment. Before (T0) and after (T1) treatment, we assessed motor performance with clinical scales and motor output with EMG-derived muscle synergies. We implemented an exploratory factor analysis (EFA) and a confirmatory factor analysis (CFA) to identify the underlying relationships among all variables, at T0 and T1, and a general linear regression model to infer any relationships between the similarity between the affected and unaffected synergies (Median-sp) and clinical outcomes at T0. Clinical variables improved with rehabilitation whereas muscle-synergy parameters did not show any significant change. EFA and CFA showed that clinical variables and muscle-synergy parameters (except Median-sp) were grouped into different factors. Regression model showed that Median-sp could be well predicted by clinical scales. The information underlying clinical scales and muscle synergies are therefore different. However, clinical scales well predicted the similarity between the affected and unaffected synergies. Our results may have implications on personalizing rehabilitation protocols.

## 1. Introduction

The execution of voluntary movement is based on the functional integration of different areas of the central nervous system (CNS) that send descending neural signals to the spinal interneurons and motoneurons to generate specific motor behaviors. Currently, the mechanisms that allow the CNS to control a large-dimensional system and coordinate many muscles consisting of thousands of motor units are still a matter of debate [1]. In describing voluntary movement, it is common to refer to the term “synergies”. However, this term may have several and different meanings, according to the context: indeed, the term *synergy* can refer to a coherent activation of a group of muscles, but it is also used with a negative connotation to describe abnormal motor patterns due to brain lesions [2]. There is also a third way of using the term synergies, commonly used to refer to a motor control model. Indeed, among the many existing models [3,4], it has been proposed that the CNS manages this complexity through a linear combination of fixed spinal modules, each one activating groups of muscles as a single functional unit, called muscle synergies [5,6,7]. Muscle synergies are obtained by decomposing surface electromyography (sEMG) into two components: vectors of fixed weights, representing the muscle synergies, and time-varying signals, representing the neural command for the synergies [8].

The activation and organization of muscle synergies are altered after stroke, causing a dysfunctional execution of voluntary movement. Early studies demonstrated that, after stroke, muscle synergies remain robust between affected and healthy arms and across subjects [9]. However, a different motor performance is observed, since abnormal motor behaviors are generated through faulty activations of the spinal modules [10,11]. The faulty activations can be generally described in terms of merging [12,13] or fragmentation [14] of the healthy muscle synergies. The degree of merging and fragmentation have been demonstrated to be proportional to the severity of motor impairment and the temporal distance from stroke onset, respectively [15].

Recent studies investigated muscle synergies as a physiological marker to assess the motor performance and recovery after stroke [14,16]. This is required because neural deficits may be masked at the functional and kinematic level by compensatory strategies, and the same motor task can be achieved by many different coordination patterns. However, it remains controversial if the use of muscle synergies can overcome these limitations, or if muscle synergies and clinical observation convey the same information on motor impairment [17]. Early studies provided evidence that muscle synergies were more adequate to capture the complexity of motor behavior than clinical scales [18]. However, some recent studies showed controversial results. In a study where muscle synergies were adopted to stratify stroke patients, synergies distributed coherently according to the Fugl-Meyer scale and Reaching Performance Scale, indicating that synergies convey similar underlying information [19]. Some other studies showed that muscle synergies and clinical scales were weakly correlated, and that stroke does not affect the inner structure of synergies, but rather their temporal recruitment [20,21]. There has also been evidence that synergies can improve in terms of their timing and organization by specific targeted therapies, including robot therapy or virtual reality treatment [15,22,23].

In rehabilitation medicine, the implication of muscle synergies should be considered as a marker of motor recovery, after a specific training for upper limb rehabilitation. Recent studies reveal there is increasing evidence demonstrating the efficacy of VR-based treatment for recovery of upper limb motor functions that facilitate the motor recovery thanks to the reinforced feedback mechanism [24]. Furthermore, it was demonstrated that after a VR treatment, patterns of cortical activation became physiologically more similar to the healthy ones, because the patterns of activations in the lesioned hemisphere were less sparse and more focused on the proper motor areas [25]. These results call into question if an underlying latent information is shared between muscle synergies and clinical scales.

The aim of this study was to identify and describe, in stroke survivors referred to upper limb treatment, whether motor output, as described by muscle synergies, and motor performance, quantified by clinical scales, convey the same information or provide a complementary one. For this purpose, a new set of variables was obtained as a combination of all the original ones by using a factor analysis. Then, we investigated whether synergies and clinical parameters belong to different components or if they convey to shared ones. In the former case, it may indicate that the two groups of variables provide different information, whereas in the latter case, there may be some muscle synergies and clinical-scale parameters that convey the same information.

## 2. Materials and Methods

### 2.1. Participants

A cohort of post-stroke patients from San Camillo IRCCS s.r.l. Hospital was recruited from a sample enrolled to participate in a multicenter clinical trial (Clinical Trial identifier: NCT03530358). We considered all patients hospitalized with diagnosis of ischemic or hemorrhagic first stroke in the territory of the middle cerebral artery (MCA).

Specifically, the following criteria were defined to recruit the patients able to perform a virtual reality treatment for upper limb motor recovery. The study included patients with a motor arm sub-score of the Italian version of the National Institute of Health Stroke Scale [26] between 1 and 3, that indicated the maintenance of residual voluntary motor activity. The following conditions were considered as exclusion criteria: (1) moderate cognitive decline defined by a Mini Mental State Examination (MMSE) [27] score lower than 20/30 points; (2) severe verbal comprehension deficits, defined by a number of errors >13 on the Token Test [28]; (3) evidence of apraxia and visuospatial neglect that could interfere with movements of the upper limb in all directions within the visual field, evaluated by neurological examination; (4) history of behavioral disorders (e.g., depression, aggressiveness, apathy) and neurological or vascular comorbidity (e.g., diabetes, myocardial infarction, Parkinson’s disease) that could affect the compliance with the rehabilitation programs.

The study was reviewed and approved by the local Ethical Committee of the IRCCS San Camillo Hospital s.r.l. All participants were adequately informed about aims and modalities of the study and provided an informed written consent.

### 2.2. Study Design

We designed a single-group longitudinal study. At the enrolment time point, a detailed review of the medical history of each patient was collected. Then, we administered a treatment consisting of 20 sessions of upper limb exercises in a virtual reality environment. To define the effect of therapy, residual motor functions were clinically and instrumentally evaluated before and after the treatment: clinical assessment consisted of standardized scales to quantify residual motor capabilities, whereas instrumental assessment consisted of the surface electromyography (sEMG) recording during the execution of motor tasks to compute muscle synergies.

#### 2.2.1. Clinical Assessment

The motor capabilities were clinically assessed with the following three outcome measures: the Modified Ashworth Scale (MAS) [29] to assess the muscle spasticity; the Fugl-Meyer Assessment scale for the upper limb (UE-FMA) [30], to determine the severity of motor impairment in hemiparetic limb, and the Reaching Performance Scale (RPS) [31] to identify and quantify movements patterns during reach-to-grasp tasks.

#### 2.2.2. sEMG Recording and Muscle Synergies

To extract the upper limb muscle synergies, we recorded the sEMG from 16 muscles from both the unaffected and the stroke-affected sides during the execution of a standard section of seven visuo-motor tasks in a virtual environment. Indeed, subjects executed seven standardized motor tasks, each repeated 10 times, by interacting with a Virtual Reality Rehabilitation System (VRRS^®^, Khymeia Group Ltd., Noventa Padovana, Italy). In VRRS^®^, the patients interacted with a VR environment by means of a 3D motion-tracking system (Polhemus 3Space FasTrack, Polhemus, Colchester, VT, USA, sampling frequency of 120 Hz) fixed on the back of the hand. At the beginning of each VRRS^®^ exercise, a trigger signal was sent to an sEMG amplifier (EMG-USB2+, OT Bioelettronica, Torino, Italy, sampling frequency of 2000 Hz) instrumented with 72001-K/12 electrodes (AMBU Neuroline, Ballerup, Denmark) to synchronize the kinematics with the sEMG [24,32,33]. The same seven tasks were proposed for both arms, except that the trajectories were mirrored according to the limb side. To facilitate the comprehension of the tasks and to reduce possible subject’s frustration, the unaffected arm was recorded first, followed by the affected arm.

Electrodes were placed according to the Surface Electromyography for the Non-Invasive Assessment of Muscles (SENIAM) recommendations for skin preparation, placement, fixation, and testing of the sensor and its connection [34]. sEMG was recorded from the following 16 muscles: triceps brachii (medial head; lateral head); biceps brachii (short head; long head); deltoideus anterior; deltoideus medius; deltoideus posterior; trapezius superior; rhomboid major; brachioradialis; supinator; brachialis; pronator teres; pectoralis major (clavicular head); infraspinatus; teres major. In the case that SENIAM recommendations were not available for a muscle, standard clinical procedures were followed [35].

The sEMG preprocessing and muscle synergies extraction followed the procedure fully described elsewhere [9,14]. Muscle synergies were extracted for the affected and unaffected arms separately. Initially, sEMG of each task were combined into an m×t matrix, where *m* indicates the number of muscles and *t* indicates the time samples. sEMG of each row in the matrix were preprocessed as follows: band-pass filtered (10–500 Hz), normalized to the unit variance, rectified, low-pass filtered to 12 Hz. Muscle synergies were extracted from 1 to 16 iteratively by decomposing the processed sEMG with the nonnegative matrix factorization (NMF) algorithm [36]. The number of synergies was chosen with a cross-validated EMG reconstruction factor R^2^ for > 90%.

From the muscle synergies of each subject, we computed the following parameters: (i) the number of synergies of the affected limb (N-aff); (ii) the number of synergies of the unaffected limb (N-ctrl); (iii) the number of synergies of the affected limb and of the unaffected limb analytically similar, with values of scalar product above 0.8 recognized as similar [37] (“Synergies shared”, N-sh); (iv) the ratio between N-sh and N-aff (Nsh-naff); (v) the ratio between N-sh and N-ctrl (Nsh-nctrl); (vi) the median scalar product between the affected and unaffected synergies (Median-sp); (vii) the mean number of unaffected synergies merging in every affected synergy (P1) (See [14]; Supporting Information).

#### 2.2.3. Rehabilitation Treatment

The rehabilitation treatment consisted of 20 sessions of one hour each, five sessions per week, 4 weeks total. To avoid discontinuity and comparable treatment intensity, at least three sessions per week were administered. Patients who performed less than 80% of the planned sessions (<16/20 sessions) were excluded from the subsequent analysis.

During each session, patients were asked to perform a defined set of exercises, including shoulder flexion–extension, abduction–adduction, internal–external rotation, circumduction, elbow flexion–extension, forearm pronation–supination, and hand–digit motion. The physical therapist was constantly present during the session, providing instructions according to specific patients’ residual abilities and needs.

### 2.3. Statistical Analysis

#### 2.3.1. Sample Characteristics

Initially, to define the sample size of the trial, we consulted previous proof-of-concept studies: they demonstrated that a sample of 20 patients are appropriate to obtain significant results [9,14]. Thus, with the aim to improve the statistical power of our analysis, we proposed to enroll 50 patients at least.

Firstly, descriptive statistics (i.e., median, interquartile range, mean, standard deviation, and percentage) were used to describe the demographic, clinical, and muscle synergies characteristics of the sample.

To verify whether there was a change in motor performance, we compared the values of the pretreatment (T0) with post-treatment (T1) clinical and instrumental variables by a paired t-test or Wilcoxon test, according to data normality distribution tested by the Shapiro–Wilk test.

Furthermore, we investigated potential associations among clinical outcomes (i.e., MAS, UE-FMA, RPS scores) and synergy parameters (i.e., N-aff, N-ctrl, N-sh, Nsh-nctrl, Nsh-naff, Median-sp, P1) by means of correlation test at T0 and T1 (i.e., Pearson correlation test or Spearman’s rank correlation test) with a significant level of correlation defined at R^2^ > 0.3.

Finally, the factorability of the data was examined by studying data sphericity with the Bartlett’s test (*p* < 0.05) [38] and data multicollinearity with the Kaiser–Meyer–Olkin measure of sampling adequacy (MSA, threshold of acceptability MSA > 0.50) [39].

#### 2.3.2. Exploratory Factor Analysis

We implemented an exploratory factor analysis (EFA) to identify the underlying relationships among all variables (EFA-All). Moreover, to investigate if the time of assessment (i.e., T0 and T1) was a parameter influencing the results, two independent models were implemented for variables acquired at T0 (EFA0) and T1 (EFA1).

To obtain each EFA model, we chose the number of latent factors [40] with the following two methods [41]: principal component analysis (PCA) [42] and principal axis factoring (PAF) [43]. We selected the most informative factors by means of the Gorsuch approach, which includes Horn’s parallel analysis, Cattell’s scree plots, and Kaiser criterion [44]. Once we found the number of factors, a common factor model was extracted with the principal axis (PA) method [45,46]. The model was rotated with oblique rotation methods (e.g., promax) [47] according to the presence of correlation between the factors [48]. Finally, we selected the most significative variables that comprised each factor according to the following criteria [43,49]: 1) factor loadings (FL) (FL > 0.3); 2) communalities, namely, common variance (h^2^ > 0.20); and 3) factors correlations (correlations r < 0.85).

#### 2.3.3. Confirmatory Factor Analysis

For each EFA model, a confirmatory factor analysis (CFA) was conducted to verify the factor structure of the observed variables (i.e., CFA-All, CFA0, CFA1). For this purpose, structural equation modeling (SEM) with a maximum likelihood estimation model and standardized coefficients (significative factor loadings FL > 0.3 or FL < −0.3) were used. Observations with missing values were excluded [50]. We assessed SEM model fitting by using the following indices [51,52]: the χ^2^ test, the comparative fit index (CFI), Tucker-Lewis index (TLI) [53,54], and root mean-squared error of approximation (RMSEA) [55,56]. The CFA model fitted the original data if the indices met the following criteria: a significant χ^2^ value indicating a bad model fit; a RMSEA value ≤ 0.05 was considered indicative of “good fit”; the CFI and TLI were considered acceptable for values >0.95 [57,58,59]. 

#### 2.3.4. General Linear Regression Model

As a final analysis, we implemented a general linear regression model to infer any potential causal relationships between the synergy parameters (dependent variable) and clinical outcomes (MAS, UE-FMA, RPS as independent variables) falling in the same factor. The statistical power of the CFA analysis was calculated with a post hoc power analysis based on the RMSEA.

The statistical significance level was set to 0.05 for all tests. All statistical analyses were performed using the free software R Studio [60].

## 3. Results

### 3.1. Sample characteristics

After the enrolment, 50 subjects completed the entire treatment. Table 1 summarizes demographic characteristics of the entire sample.

After inferential analysis, two out three clinical outcomes improved significantly: the UE-FMA score improved by 6% (T0, UE-FMA mean = 117.2; T1, UE-FMA mean = 124.26), and the RPS score improved by 4% (T0, RPS mean = 25.4; T1, RPS mean = 26.46). Conversely, synergies parameters revealed no significant change after the treatment. Table 2 reports the clinical outcomes and parameters related to the muscle synergies.

**Figure 1:**
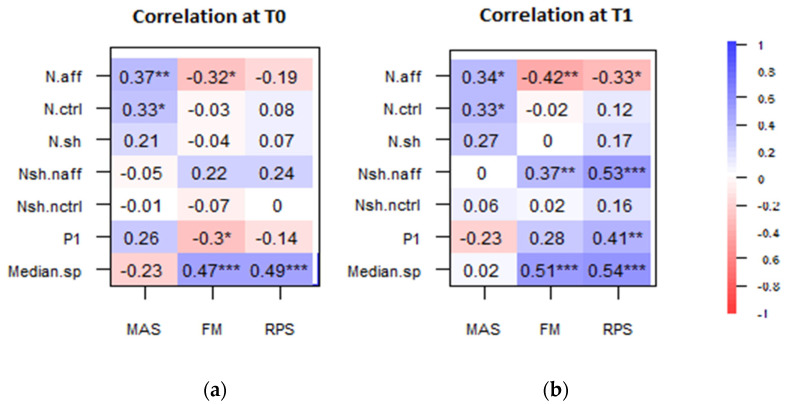
Correlation between clinical outcomes and parameters related to synergies at T0 (**a**) and at T1 (**b**). Significant correlation indices are indicated with * *p* < 0.05, ** *p* < 0.01, and *** *p* < 0.001, respectively.

Moreover, correlation analysis showed that some of the clinical scales were significantly correlated with some muscle synergy parameters. Specifically, there was a positive correlation between MAS and N-aff (R^2^ = 0.37 at T0; R^2^ = 0.34 at T1). Moreover, the clinical scale UE-FMA and RPS correlated positively both with Nsh-naff after treatment (UE-FMA, R^2^ = 0.37 and RPS, R^2^ = 0.53 at T1) and with Median-sp (UE-FMA, R^2^ = 0.47 and RPS, R^2^ = 0.49 at T0) (UE-FMA, R^2^ = 0.51 and RPS, R^2^ = 0.54 at T1). Figure 1 summarizes the correlation coefficients between clinical outcomes and synergies parameters, at T0 and T1, separately.

Both the Bartlett’s test of sphericity ($\chi^{2}$ = 1396.80, df = 190, *p* < 0.001) and the Kaiser–Meyer–Olkin test (MSA = 0.62) indicated that the correlation matrix was factorable. Then, we proceeded with the factor analysis at T0, T1, and with all the variables.

### 3.2. Exploratory Factor Analysis

#### 3.2.1. Exploratory Factor Analysis with All Variables

At first, we implemented the exploratory factorial analysis on the whole sample (EFA-All) and we obtained structures with three to five factors (Supplementary Material, Figure S1a). All these factor solutions were sequentially examined, with a total explained variance equal to 66%. Specifically, one factor was linked to the clinical variables (both pre- and post-treatment) and two factors, linked to the parameters of the synergies derived from pre- and post-treatment EMGs, respectively.

Table 3 reports the loadings and the corresponding communalities of the EFA-All. It can be observed that Factor 1 was linked to the clinical variables (both pre- and post-treatment) and Median-sp-T0. Factor 2 and Factor 3 were linked to parameters of the synergies only.

Factor correlations were r = −0.30 between Factor 1 and Factor 2, r = 0.145 between Factor 1 and Factor 3, and r = 0.37 between Factor 2 and Factor 3.

#### 3.2.2. Exploratory Factor Analysis with T0 Variables

Secondly, we implemented an EFA model with two factors for the variables at T0 time point (EFA0). The factor structure of the sample indicated the presence of more than one unique factor, suggesting that two factors should be retained (Supplementary Material, Figure S1b). The correlation between the two factors was very low (r = −0.0013); therefore, a promax oblique rotation method was used. Table 4 reports the loadings of the factor matrix.

These factors collectively accounted for 70.2% of the variance in the responses. Factor correlation was r = −0.14.

#### 3.2.3. Exploratory Factor Analysis with T1 Variables

Finally, we implemented an EFA model with two factors for the variables at T1 time point (EFA1). The factors were rotated with promax oblique rotation methods as the correlation between the two factors was r = 0.023 (Supplementary Material, Figure S1c). Table 5 reports the loadings of the factor matrix.

These factors collectively accounted for 76.2% of the variance in the responses and they had a factor correlation of r = −0.068.

### 3.3. Confirmatory Factor Analysis 

After EFA analysis, we proceeded to confirmatory factor analysis, for all sample variables and then for variables at T0 and T1.

#### 3.3.1. Confirmatory Factor Analysis with All Variables

In the CFA analysis carried out on all variables (CFA-All), a latent three-factor model was specified, as suggested by the results obtained in the EFA-All analysis.

**Figure 2:**
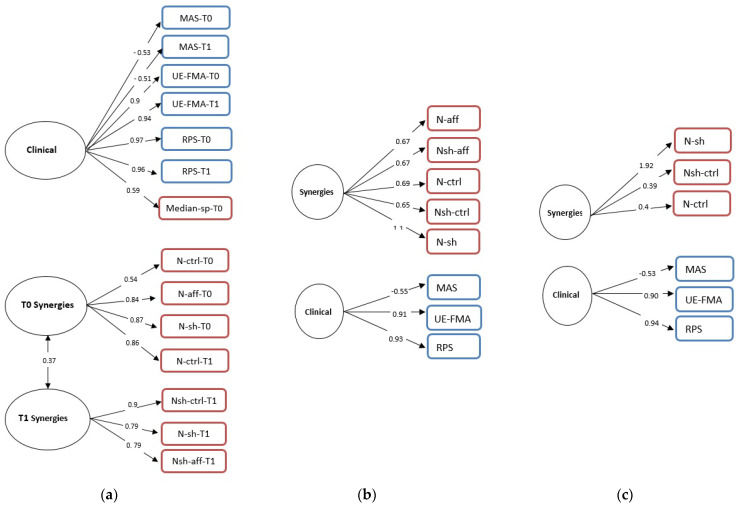
Confirmatory factor analysis for all variables (**a**) at T0 (**b**) and at T1 (**c**). Single-headed arrows indicate direct relationships. The numbers on each represent standardized factor loadings ranging from 1.0 to −1.0. Squares represent measured variables and circles represent latent factors. The figures in blue represent the clinical variables, while those in red represent the synergies parameters. The double-headed arrows represent correlations between the factors.

Based on the content of their variables, we named the three factors clinical scale, T0 synergies, and T1 synergies (Figure 2a).

The CFA-All model indicated the presence of a correlation between two factors, the T0 synergies and T1 synergies factors (r = 0.37), while no correlations related to the clinical scale factor were detected. Furthermore, all factor loadings were significant. Goodness-of-fit statistics demonstrated that all indices were outside the set cut-offs: RMSEA index between 0.30 and 0.36 and a value of χ^2^ = 442.84, with df = 74 and *p* = 0.000. The values of CFI and TLI were 0.55 and 0.45, respectively.

#### 3.3.2. Confirmatory Factor Analysis T0 Variables

The EFA0 suggested a two-factor solution, and we estimated a latent two-factor model (CFA0). According to the content of their items, we named the two factors as clinical scale and synergies parameters (Figure 2b). After estimating the model, goodness-of-fit statistics were obtained. All FL were significant, but the model demonstrated that all indices were outside the set cut-offs, with RMSEA index between 0.45 and 0.57 and χ^2^ = 255, with df = 19 and *p* = 0.000. Moreover, the values of CFI and TLI were 0.57 and 0.37, respectively.

#### 3.3.3. Confirmatory Factor Analysis T1 Variables

The EFA1 suggested a two-factor solution, and we estimated a latent two-factor model (CFA1). According to the content of their items, we named the two factors as clinical scale and synergy parameters (Figure 2c). After estimating the model, it did not show a very good fit, with an RMSEA index between 0.11 and 0.29 and χ^2^ = 23.58, with df = 8 and *p* = 0.003. Moreover, the values of CFI and TLI were 0.95 and 0.91, respectively.

### 3.4. General Linear Regression Models

To investigate the reason why the Median-sp at T0 was associated with the clinical factor in both EFA-All and CFA-All, we estimated a general linear regression model with the Median-sp as dependent variable and the clinical variables (i.e., MAS, RPS, UE-FMA) at T0 as independent variables.

In the general linear regression model, the T0 variable Median-sp was significantly associated with RPS at T0 ($\hat{\beta }\;$= 0.002, *p* < 0.001). The model determination coefficient was R^2^ = 0.97.

## 4. Discussion

In the present study, we investigated the statistical relationships among the clinical variables and muscle synergy parameters in a cohort of post-stroke patients enrolled in a specific treatment for upper limb motor recovery provided in a virtual environment. Specifically, the main objective of this study was to identify whether motor output, as described by muscle synergies parameters recorded using sEMG, and motor performance, quantified by clinical scales, convey the same information or provide a complementary one.

In our study, the pre- and post-treatment analysis evidenced a significant improvement in almost all clinical outcomes, whereas no significant differences in muscle synergies parameters were observed. This suggests that they have a different sensitivity to the recovery level after stroke, and that the number of muscles synergies and merging alone are not sensitive enough to describe the effectiveness of treatment. This seems to be in contrast with some previous studies, where changes in the number of synergies and their structure indicated improvement of motor control and movement quality. However, it was limited to patients with low level of residual motor functions [15,61].

There may be several mechanisms that could better describe how muscle synergies change after motor therapy. For instance, rather than counting the number of muscle synergies, it has been shown that modifications of clusters and shifting from one cluster to another can provide insights for assessing the efficacy of the therapy [62]. After stroke, functional and structural recovery processes take place within the brain. Since these processes are mainly related to the reorganization of cortical maps [63], we may hypothesize that changes in the modulation of synergies may be associated with these mechanisms of neural plasticity. This could trigger changes in muscle coactivation within synergies, resulting in synergy merging, for instance [64]. Indeed, it was suggested that the merging phenomena of muscle synergies may depend on neural changes at the cortical level or at the level of the brainstem in the spinal cord [14].

Despite that clinical and instrumental assessments provided different information in terms of motor recovery, some strong relationships emerged between some muscle-synergy parameters and clinical scales. Indeed, the correlation analysis indicated that the number of muscle synergies of the affected limb was positively correlated with clinical outcomes: the higher the N-aff, the higher the level of spasticity to upper limb muscles (i.e., MAS). Conversely, the higher the N-aff, the lower the level of motor ability (i.e., UE-FMA and RPS). In line with our findings, Pan et al. [13] found that muscle synergies were significantly positively correlated with the Brunnstrom stage (R^2^ = 0.52, *p* < 0.01). This is in good agreement with our study, because the Brunnstrom scale describes the stages of stroke recovery by a progressive decrease in spasticity. Furthermore, there was a strong positive correlation between the Median-sp values and the motor ability of the patients (i.e., UE-FMA and RPS). Since Median-sp describes the similarity between the affected and unaffected synergies, it seems to provide some useful and objective information about the degree of “true recovery” of the paretic arm (i.e., the extent to which the original muscle coordinative structures are restored) after intervention. Moreover, the correlation index increased after the treatment (i.e., T1 assessment), meaning that, after motor treatment, Median-sp is more informative about the motor performance.

The second objective of the study was to group all the variables into one or more clusters and to describe the nature of the underlying relationships among variables as described by the latent factors. More precisely, we used EFA and CFA to explore the information shared between muscle synergies and clinical scales of stroke survivors referred to upper limb treatment.

The EFA model we implemented with all variables (i.e., EFA-All) identified three factors: one linked to the clinical variables (both pre- and post-therapy), and two linked to the pre- and post-treatment parameters of the synergies, respectively. Median-sp was the only muscle-synergy parameter which was associated with the cluster of clinical scales, and thus with the level of a patient’s motor performance. This achievement confirms the correlation results, and it represents a potential continuous index of similarity that can provide information also from a clinical point of view. Our finding that clinical variables and muscle-synergy parameters were mostly linked to separable factors argues that muscle synergies and clinical variables provided complementary information, both related to the motor ability of the patient.

The subsequent models, EFA0 and EFA1, highlighted the same structure of EFA-All, with the variables clustered according to their nature: clinical scales and synergy parameters. It should be noted that a synergy parameter (N-ctrl T1), despite referring to post treatment outcome, is attributed to the pretherapy cluster. This is an expected result: since N-ctrl was obtained from the healthy limb, it did not vary due to the motor therapy. On the other hand, in the CFA-All model, the variables were not distributed clearly among the factors according to their nature, as we would expect. Similar to the EFA-All model results, in the clinical factor, there was a parameter of the Median-sp-T0 synergies, while in the two factors related to the synergies (T0 synergies and T1 synergies) there were parameters that did not follow the temporal subdivision of their nature. Moreover, in CFA0 and CFA1, the variables were not all represented by latent factors. Indeed, both in the CFA0 and in the CFA1 models, the clinical factor collects all the clinical variables considered (i.e., MAS, UE-FMA, and RPS). 

By considering the differences between CFA0 and CFA1 models, it was highlighted how, after the therapy, the parameters linked to the stroke-affected limb (i.e., N-aff and N-sh-aff) disappeared, probably because after the therapy the differences between healthy limb and affected limb were less marked, and therefore the affected limb no longer provided information. 

Since in both EFA-All and CFA-All models, Median-sp-T0 was the only synergy parameter with an underlying structure in common with the clinical scales, a regression model was estimated to determine whether there is a causal relationship between the similarity parameter (Median-sp) (dependent variable) and all clinical variables (independent variables) at the same time point, that is, the pretreatment evaluation (i.e., T0). Our model evidenced a relationship between the similarity of affected and healthy synergies at the beginning of the treatment and the upper limb movement quality during a reach-to-grasp target, assessed by RPS [65]. Indeed, the presence of the RPS scale in the model is consistent with the indication that the reaching movement may be the best predictor of motor recovery. Recently, Pan et al. defined that severe patients had the lowest range of motion and speed during reaching movement. Specifically, they found three muscle synergies that may explain reaching movement. Moreover, severe patients changed one of these muscle synergies; meanwhile, the mild-to-moderate patients were more similar to the control template [66]. Thus, individualized training may be developed to make the patients’ features more similar to the ones in control subjects so as to improve similarity values (i.e., Median-sp) [67].

The present study has several limitations that should be addressed in future research. In terms of generalizability, the relatively small sample size used to conduct the factor analysis (i.e., both EFA and CFA methods) should not be considered to obtain valuable results as good-of-fitness index [68]. For this reason, the goodness-of-fit statistics for all CFA models showed that none of them had good overall fit, with RMSEA never dropping below 0.11 and CFI and TLI remaining relatively low, despite a high post hoc statistical power (1 − beta > 0.99). Moreover, to obtain an effect on the synergies, we need to test a longer treatment period or to tailor the upper limb treatment based on the stratification of patients. Indeed, the sample of patients was not homogeneous in terms of timing from lesion and initial level of motor impairment. Actually, more investigation is needed concerning which neurophysiological parameters may help classify patients based on different recovery potential [69,70]. In our study, our results demonstrate that muscle-synergy parameters showed a potential to contributing to discriminate between patients with different recovery potential: the relationship between neurophysiological parameters (i.e., Median-sp) and clinical variables at the beginning of therapy gave some indication about the potential patient-tailored treatment. More trials will be needed to define the real contribution of muscle-synergy parameters to distinguish between fitters and no-fitters of reactive neurobiological recovery [66,71]. Moreover, we may consider using the similarity parameter (i.e., Median-sp) to build patient-specific prediction models to improve clinical decisions, and, ultimately, recovery and outcome after stroke [72].

Finally, the types of movements and the kinematics were not considered in our study, making other biomechanical interpretation of our results possible.

## 5. Conclusions

In our study, we investigated whether there is a relationship between clinical scales and muscle-synergy parameters in individuals with stroke who underwent a specific upper limb motor training. Specifically, after statistical analysis, we found that there exists a relationship between the similarity of muscle synergy parameters of the affected and unaffected limb and clinical variables, in particular at the beginning of the therapy. The correlation found between Median-sp and clinical variables indicates that there is a related, but complementary, information provided by both different type of parameters. Finally, future analyses may be conducted to investigate the use of similarity parameter as a biomarker of different levels of motor impairment to tailor the upper limb motor treatment to stroke survivors.

## Figures and Tables

**Table 1:** Demographic and clinical characteristics of the patients.

Patients (N = 50)	
Sex, males/females, n (%)	33 (66%)/17 (34%)
Age, years, mean ± SD	63.62 ± 12.29
Diagnosis, ischemic/hemorrhagic, n (%)	45 (90%)/5 (10%)
Hemisphere, left/right, n (%)	25 (50%)/25 (50%)
Time-stroke, months, mean ± SD	6.99 ± 13.07
0–3 months, n, mean ± SD	15, 2.32 ± 0.42
3–6 months, n, mean ± SD	17, 4.25 ± 0.87
>6 months, n, mean ± SD	18, 20.61 ± 19.83

Values are expressed as mean ± standard deviation (SD) for quantitative measures, and frequency percentages (%) for all discrete variables.

**Table 2:** Clinical outcomes and parameters related to synergies.

**Clinical** **Parameters**	**T0**		**T1**		***p* Value**
	**Median [IQR]**	**Mean ± SD**	**Median [IQR]**	**Mean ± SD**	
MAS	1 [2.75]	1.92 ± 2.69	0.5 [2]	1.60 ± 2.44	0.098
UE-FMA	125.5 [34.75]	117.20 ± 24.57	131.5 [33.25]	124.26 ± 25.41	<0.001 *
RPS	30 [6]	24.4 ± 11.19	17 [6]	26.46 ± 12.25	<0.001 *
**Synergies** **Parameters**	**T0**		**T1**		***p* Value**
	**Median [IQR]**	**Mean ± SD**	**Median [IQR]**	**Mean ± SD**	
N-aff	8 [1]	8.42 ± 1.40	8 [2]	8.20 ± 1.47	0.289
N-ctrl	8 [2]	7.86 ± 1.31	8 [1.75]	7.84 ± 1.22	0.855
N-sh	6 [2]	6.24 ± 1.39	6 [2]	6.12 ± 1.36	0.456
Nsh-naff	0.75 [0.13]	0.74 ± 0.12	0.78 [0.22]	0.75 ± 0.13	0.616
Nsh-nctrl	0.79 [0.16]	0.79 ± 0.12	0.78 [0.14]	0.78 ± 0.12	0.432
Median-sp	0.93 [0.04]	0.92 ± 0.04	0.94 [0.05]	0.93 ± 0.03	0.056
P1	1.19 [0.58]	1.25 ± 0.39	1.24 [0.44]	1.24 ± 0.34	0.913

Values are expressed as median [IQR] and mean ± SD; IQR = interquartile range; SD = standard deviation; * *p* values < 0.05; Wilcoxon test.

**Table 3:** EFA-All, with promax rotation for all variables.

Outcome	Factor 1	Factor 2	Factor 3	h^2^
MAS-T0	−0.579			0.528
UE-FMA-T0	0.914			0.839
RPS-T0	0.948			0.885
MAS-T1	−0.554			0.467
UE-FMA-T1	0.988			0.920
RPS-T1	0.918			0.882
Median-sp-T0	0.616			0.441
N-aff-T0		0.913		0.848
N-ctrl-T0		0.922		0.669
N-sh-T0		0.972		0.849
Nsh-ctrl-T0		0.301		0.218
N-ctrl-T1		0.537		0.415
N-sh-T1			0.780	0.847
Nsh-aff-T1			0.881	0.769
Nsh-ctrl-T1			0.921	0.687
Median-sp-T1			0.589	0.503
**% variance of the factor**	33.7%	16.5%	15.9%	

Table shows the factor loadings for the 3 factors and the communalities for each variable (h^2^).

**Table 4:** EFA0, with promax rotation for variables at T0.

Outcome	Factor 1	Factor 2	h^2^
MAS	−0.618		0.420
UE-FMA	0.847		0.705
RPS	0.886		0.775
N-aff		0.631	0.759
Nsh-aff		0.811	0.751
N-ctrl		0.674	0.538
Nsh-ctrl		0.811	0.751
N-sh		1.067	1.157
**% variance of the factor**	39.3%	30.9%	

Table shows the factor loadings for Factor 1 and Factor 2, the communalities for each variable (h^2^), and the percentage of variance explained by each factor (%).

**Table 5:** EFA1, with promax rotation for variables at T1.

Outcome	Factor 1	Factor 2	h^2^
MAS		−0.562	0.627
UE-FMA		0.889	0.786
RPS		0.948	0.948
N-ctrl	0.550		0.310
Nsh-ctrl	0.505		0.255
N-sh	1.390		1.933
**% variance of the factor**	42.8%	33.4%	

Table shows the factor loadings for Factor 1 and Factor 2, the communalities for each variable (h^2^), and the percentage of variance explained by each factor (%).

## Data Availability

The clinical protocol of this study is reposed and publicly available at the following address: https://www.clinicaltrials.gov/ct2/show/NCT03530358. The data presented in this study are available on request from the corresponding author.

## Supplementary Materials

The following are available online at https://www.mdpi.com/article/10.3390/s21238002/s1, Supplementary file “Supplementary Materials.docx” contains the Cattell’s scree plots based on both PAF and PCA for the EMA models presented in the main text. Figure S1. Cattell’s scree plot based on both PAF and PCA for EFA-All (**a**), EFA0 (**b**) and EFA1 (**c**). Cattell’s rule states that number of factors and components, corresponding to eigenvalues to the up of the straight line should be retained. Abbreviations: FA = Factor Analysis; PC = Principal Component.

## Abbreviations

CNS	Central nervous system
sEMG	Surface electromyography
MCA	Middle cerebral artery
MMSE	Mini Mental State Examination
MAS	Modified Ashworth Scale
UE-FMA	Upper Extremity Fugl-Meyer Assessment Scale
RPS	Reaching Performance Scale
VRRS	Virtual Reality Rehabilitation System
SENIAM	Surface Electromyography for the Non-Invasive Assessment of Muscles
NMF	Nonnegative matrix factorization
N-aff	Number of affected synergies
N-ctrl	Number of unaffected synergies
N-sh	Number of shared synergies
Nsh-naff	Percentage of synergies shared in the affected arm
Nsh-nctrl	Percentage of synergies shared in the unaffected arm
Median-sp	Median of the calar product between the affected and unaffected arm
P1	Merging parameter
T0	Pretherapy variable
T1	Posttherapy variable
R2	Correlation coefficient
P	P-value
MSA	Measure of sampling adequacy
EFA	Exploratory factor analysis
EFA0	Exploratory factor analysis at T0
EFA1	Exploratory factor analysis at T1
EFA-All	Exploratory factor analysis with all variables at T0 and T1
PCA	Principal component analysis
PAF	Principal axis factoring
PA	Principal axis
FL	Factor loadings
h2	Communalities
r	Factors correlation coefficient
CFA	Confirmatory factor analysis
CFA0	Confirmatory factor analysis at T0
CFA1	Confirmatory factor analysis at T1
CFA-All	Confirmatory factor analysis with all variables at T0 and T1
CFI	Comparative fit index
χ^2^	Chi-squared
TLI	Tucker–Lewis index
RMSEA	Root mean-squared error of approximation
Df	Degrees of freedom
SD	Standard deviation
IQR	Interquartile range
FA	Factor analysis
PC	Principal component
$\hat{\beta }$	Estimate regression coefficient.
