# Supplementary material for: Muscle Synergies and Clinical Outcome Measures Describe Different Factors of Upper Limb Motor Function in Stroke Survivors Undergoing Rehabilitation in a Virtual Reality Environment
Source: Sensors (Basel). 2021 Nov 30;21(23):8002. doi: 10.3390/s21238002 (PMC8659727; doi:10.3390/s21238002)
Supplement: Supplementary file 1 [file sensors-21-08002-s001.zip › sensors-1464780-supplementary.pdf]

sensors

MDPI

Article

Muscle Synergies and Clinical Outcome Measures Describe Different Factors of Upper Limb Motor Function in Stroke Survivors Undergoing Rehabilitation in a Virtual Reality Environment

Lorenza Maistrello [1], Daniele Rimini [2,*], Vincent C.K. Cheung [3], Giorgia Pregnolato [1] and Andrea Turolla [1]

[1] Laboratory of Rehabilitation Technologies, IRCCS San Camillo Hospital, 30126, Venice, Italy; lorenza.maistrello@hsancamillo.it (L.M.); giorgia.pregnolato@hsancamillo.it (G.P.); andrea.turolla@hsancamillo.it (A.T.)

[2] Medical Physics Department - Clinical Engineering, Salford Care Organisation, Salford, M6 8HD, United Kingdom; daniele.rimini@nca.nhs.uk (D.R.)

[3] School of Biomedical Sciences, The Chinese University of Hong Kong, Hong Kong, China; vckc@cuhk.edu.hk (V.C.K.C.)

* Correspondence: daniele.rimini@nca.nhs.uk; Tel.: +44 61620(64859)

Citation: Maistrello, L.; Rimini, D.; Cheung, V.C.K.; Pregnolato, G. Turolla, A.; Muscle Synergies and Clinical Outcome Measures Describe Different Factors of Upper Limb Motor Function in Stroke Survivors Undergoing Rehabilitation in a Virtual Reality Environment. *Sensors* **2021**, *21*, 8002. https://doi.org/10.3390/s21238002

Academic Editor: Francesco Di Nardo, Valentina Agostini and Silvia Conforto

Received: 30 October 2021
Accepted: 26 November 2021
Published: date

Publisher's Note: MDPI stays neutral with regard to jurisdictional claims in published maps and institutional affiliations.

Copyright: © 2021 by the authors. Submitted for possible open access publication under the terms and conditions of the Creative Commons Attribution (CC BY) license (https://creativecommons.org/licenses/by/4.0/).

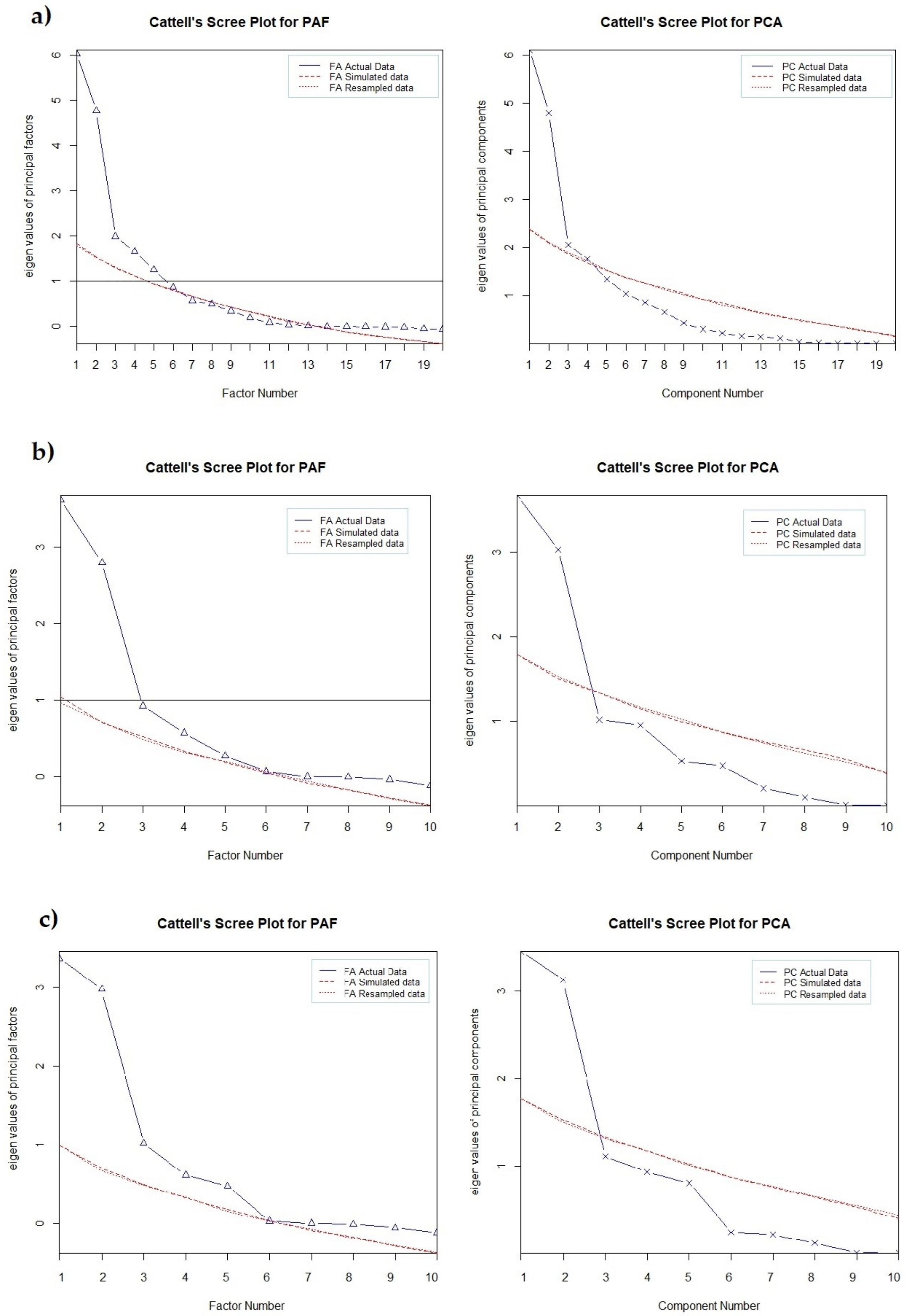

Figure S1. Cattell's scree plot based on both PAF and PCA for EFA-All (**a**), EFA0 (**b**) and EFA1 (**c**). Cattell's rule states that number of factors and components, corresponding to eigenvalues to the up of the straight line should be retained. Abbreviations: FA = Factor Analysis; PC = Principal Component.
